# Facile Fabrication of Durable Superhydrophobic Films from Carbon Nanotube/Main-Chain Type Polybenzoxazine Composites

**DOI:** 10.3390/polym11071183

**Published:** 2019-07-14

**Authors:** Chih-Feng Wang, Wen-Ning Wang, Ching-Hsuan Lin, Kuo-Jung Lee, Chien-Chieh Hu, Juin-Yih Lai

**Affiliations:** 1 Graduate Institute of Applied Science and Technology, National Taiwan University of Science and Technology, Taipei 106, Taiwan; 2 Department of Materials Science and Engineering, I-Shou University, Kaohsiung 840, Taiwan; 3 Department of Chemical Engineering, National Chung Hsing University, Taichung 402, Taiwan; 4 Department of Chemical Engineering, National Taiwan University of Science and Technology, Taipei 106, Taiwan; 5 R&D Centre for Membrane Technology, Chung Yuan University, Taoyuan 320, Taiwan

**Keywords:** carbon nanotubes, polybenzoxazine, superhydrophobic, durability

## Abstract

Superhydrophobic materials have immense applications in the fields of industry and research. However, their durability is still a cause for concern. A facile method for preparing durable superhydrophobic films from carbon nanotubes (CNTs) and the main-chain type polybenzoxazine precursors is reported herein. We used probe ultrasonicator to prepare CNT/polybenzoxazine coatings. Compared with the general sonicating dispersion process, the dispersion time was greatly reduced from a few hours to 5 minutes and the prepared suspension exhibited film-forming characteristics well. The CNT/polybenzoxazine films, which do not contain any fluorinated compounds, exhibit remarkable durability against thermal treatment, organic solvents, corrosive liquids, and sandpaper abrasion, while retaining their superhydrophobicity. Furthermore, these CNT/polybenzoxazine films also showed durable superhydrophobicity after ultraviolet (UV) irradiation for 100 h. This CNT/polybenzoxazine film can be readily used for practical applications to make durable superhydrophobic coatings.

## 1. Introduction

Superhydrophobicity is found in many organisms, for instance, leaves of lotus, leaves of rice, butterfly wings, water strider legs, and mosquito eyes [1,2,3,4]. This phenomenon has gained increasing attention from many scientists since the last century. The basis of the solid surface repellency towards liquid is inevitable in the applications of solid surface [5]. In general, the nature of the liquid repellency towards solid surface was determined by the topographical microstructure and surface chemical composition of both phases. The surfaces possessing this property depict a high water contact angle (over 150°) and a small sliding angle (below 10°).According to the Wenzel model, the surface area of a hydrophobic solid was increased by surface roughness resulting in increased hydrophobicity [6]. Additionally, per the Cassie–Baxter model, the superhydrophobic behavior was caused by tiny air pockets trapped in the grooves underneath the liquid [7]. Researchers have known that the superhydrophobic surface materials should coexist, based on the phenomenon observed in nature; these materials are characterized by both micro and nanoscale surface roughness and low surface-freeenergy. These surfaces have wide applications in various fields such as oil/water separation, and dragging reduction, and are used in the manufacture of self-cleaning paints, microfluidic systems, medical equipment, and anticorrosive materials, amongst others.

Despite the advantage of the superhydrophobic materials in large-scale industrial applications, the durability of the surface is a major issue limiting these applications. Surfaces with high roughness usually have poor mechanical strength compared to a smooth surface. Toaddress these issues, different methods have been suggested to enhance the durability of superhydrophobic materials [8,9,10,11,12,13,14]. Chen et al. fabricated superhydrophobic surfaces through a straightforward spraying process with the suspension of perfluorosilane-modified CaCO3 nanoparticles [15]. These superhydrophobic surfaces exhibited tremendous resistance against a sandpaper abrasion test and knife scratch. Li and coworkers applied a rapid ultraviolet (UV) cross-linking method to fabricate stretchable surfaces exhibiting excellent superhydrophobicity and self-cleaning ability when encountered with mechanical abrasion and stretching damage [16]. Peng et al. synthesized an all-organic superhydrophobic nanocomposite coating that preserved its non-wetting properties under a variety of harshmechanical and chemical environments [17].

Owing to the excellent chemical, electronic, and mechanical properties of carbon nanotubes (CNTs), these are being increasingly used in several research fields in science and technology. Similarly, the unique properties of polybenzoxazines such as high glass-transition temperature, high moduli, and low water absorption have made these resins effective thermosetting polymers [18]. It is widely accepted that the physical interactions (especially hydrogen bonding) between polybenzoxazine polymer chains are responsible for their various attractive properties. In our recent studies, it has been reported that extremely low surface-freeenergies, even lower than those of pure Teflon, could be found on the polybenzoxazine surfaces, owing to their strong intramolecular hydrogen bonding [19]. Fluoropolymers and polybenzoxazines both have low surface-free energies. However, the polybenzoxazines are cheaper to synthesize and easier to process.

Herein, we report a straightforward method to fabricate durable superhydrophobic films through CNT and main-chain type polybenzoxazine precursor. Thus, multifunctional CNT/polybenzoxazine films were prepared with readily available raw materials under ambient conditions using lab equipment. The as-prepared superhydrophobic films possessed good film-forming characteristics and could pass the3H pencil hardness tests. Moreover, these films exhibit remarkable durability against thermal treatment, organic solvents, corrosive liquids, and sandpaper abrasion, while retaining their superhydrophobicity. Furthermore, these CNT/polybenzoxazine films also showed durable superhydrophobicity after UV irradiation for 100 h.

## 2. Experimental Section

Multiwalled CNT (length > 100 μm; average diameter:20–40 nm) were purchased from Golden Innovation Business Co., Ltd. (Taipei, Taiwan) The main-chain type polybenzoxazine precursor P(B-oda) was prepared according to our previous report [20]. The chemical structure of P(B-oda) is shown in Figure S1.

**Scheme 1:**
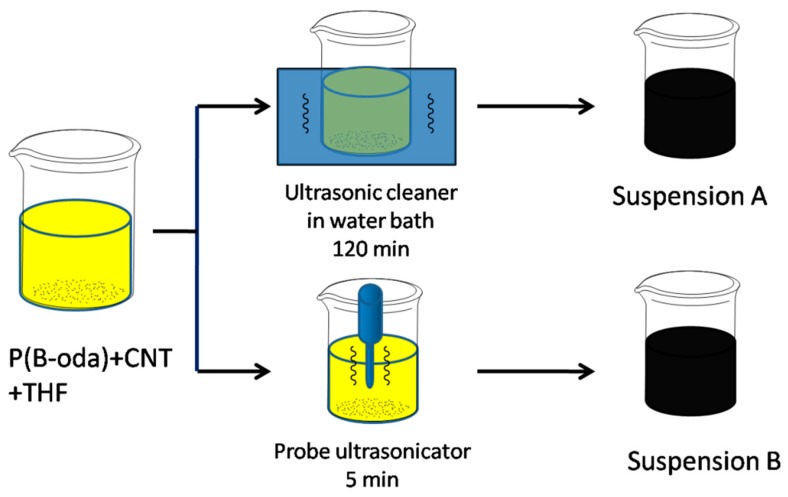
Scheme of the preparation of carbon nanotube (CNT)/main-chain type polybenzoxazine precursor suspensions.

We used a deposition method to prepare superhydrophobic coatings on a glass slide. In brief, P(B-oda) (10 mg) was dissolved in tetrahydrofuran (THF, 10 mL) to prepare P(B-oda) solution; the solution was then filtered through a polytetrafluoroethene (PTFE) syringe filter (0.2 μm). This was followed by the addition of CNTs (20 mg) to the P(B-oda) solution. The as-prepared solution was dispersed using anultrasonic cleaner (DC150H, Delta, New Taipei City, Taiwan) in water bath for 120 min (suspension A) or directly dispersed through probe ultrasonicator (Q125, Qsonica, Newtown borough, CT, USA) for 5 min (suspension B) (Scheme 1). The resulting CNT/P(B-oda) suspension was poured onto a clean glass slide in an aluminum container. The sample was dried at room temperature for 8 h. Finally, the sample was cured in an oven at 240 °C for 1h. Samples prepared using suspension A and suspension B were termed as sample A and sample B, respectively.

The microstructure of the CNT/polybenzoxazine film was characterized by using a scanning electron microscope (SEM; Hitachi S-4700, Tokyo, Japan). We used a contact angle goniometer (FDSA MagicDroplet-100, Sindatek Instruments Co., Ltd., Taipei, Taiwan) to measure contact and sliding angles. The static water contact angle and the sliding angle were obtained utilizing 5 and 10 μL drops, respectively. Thermogravimetric analysis (TGA; TA Instruments Q50, New Castle, DE, USA) was conducted under N_2_, temperature up to700 °C, and a heating rate of 20 °C min^−1^.The superhydrophobic films were immersed in organic solvents for 6 h to study the film’s durability to organic solvents. The contact angle was measured on the dried film. Superhydrophobic film’s pencil hardness was measured by using the method described in ASTMD3363. For the UV irradiation tests, five 8 W low-pressure mercury lamps (λ = 256 nm) were used; the distance between UV light source and the superhydrophobic film was approximately 12cm.

## 3. Results and Discussion

In recent years, main-chain-type polybenzoxazines have emerged as intriguing materials. In our previous study, we revealed that cured P(B-oda) films exhibit unusual flexibility, high glass transition temperature (*T*_g_ = 303 °C), and low surface free energy (17.9 mJ m^−2^) [20].The contact angle of water droplet on the cured P(B-oda) coating was 102 ± 2°, although it considered beinghydrophobicbut cannot be classified as superhydrophobic.

**Figure 1:**
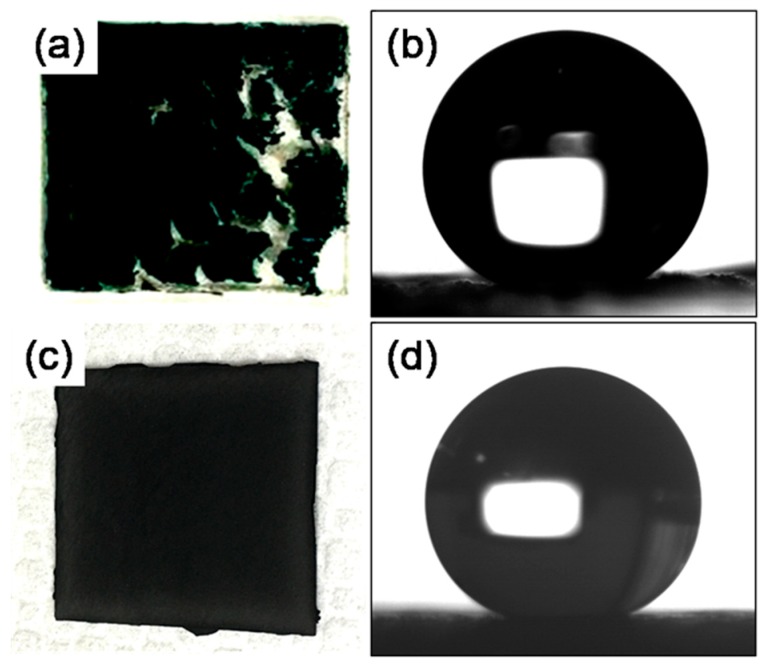
(**a**) Photograph of sample A. (**b**) Profile of a water drop on sample A. (**c**) Photograph of sample B. (**d**) Profile of a water drop on sample B.

The combination of P(B-oda)with CNTs was used to prepare superhydrophobic coatings on the glass slides. The CNT/polybenzoxazine films prepared in this study, and classified as Sample A and Sample B based on the dispersion method (Scheme 1) are shown in Figure 1a,b. Figure 1c,d show the photographs and the profile of a water drop on as-prepared CNT/polybenzoxazine surfaces. Sample A and sample B both possess superhydrophobic properties. The water contact angles are 162° and 167° for samples A and B, respectively, while the sliding angles are 6° and 3°, respectively. However, sample B possesses better film-forming ability than sample A, as it was observed that CNT/polybenzoxazine films prepared using suspension A could not completely cover the glass substrate.

**Figure 2:**
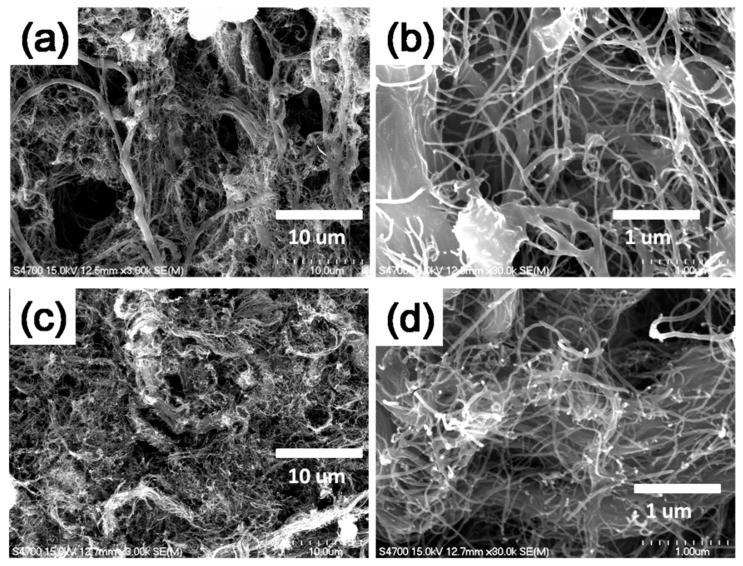
(**a**) Large-area scanning electron microscope (SEM) image of sample A. (**b**) Enlarged view of (**a**). (**c**) Large-area SEM image of sample B. (**d**) Enlarged view of (**c**).

Figure 2a–d display top-view SEM images of CNT/polybenzoxazine films. Such morphology drastically increases the surface roughness and yields a composite interface to facilitate the air trapped in grooves below the liquid, thereby enhancing superhydrophobicity [21]. Nevertheless, from Figure 2a, it is indicated that the CNTs were not dispersed well using the ultrasonic cleaner in a water bath, even after treatment for 120 min; a considerable number of CNT bundles in CNT/polybenzoxazine films were detected. The CNT was not fully covered and connected well through polybenzoxazines in these films (Figure 2b). Figure 2c,d present top-view SEM images of sample B. Via using a probe ultrasonicator with a relatively short dispersion time (5 min), we succeeded in preparing uniform CNT/polybenzoxazine films (Figure 2c). Typical branch-like CNT nanostructures conjugated to the polybenzoxazine films, as observed from Figure 2d. To further analyze the differences in robustness between samples A and B, we performed the pencil scratch test. Based on the results of this test, we found that the hardness of the sample B was 3H, while the hardness of sample A was only 6B. This indicated that improved dispersion of CNT in CNT/polybenzoxazine films greatly enhanced their film-forming ability and hardness.

**Figure 3:**
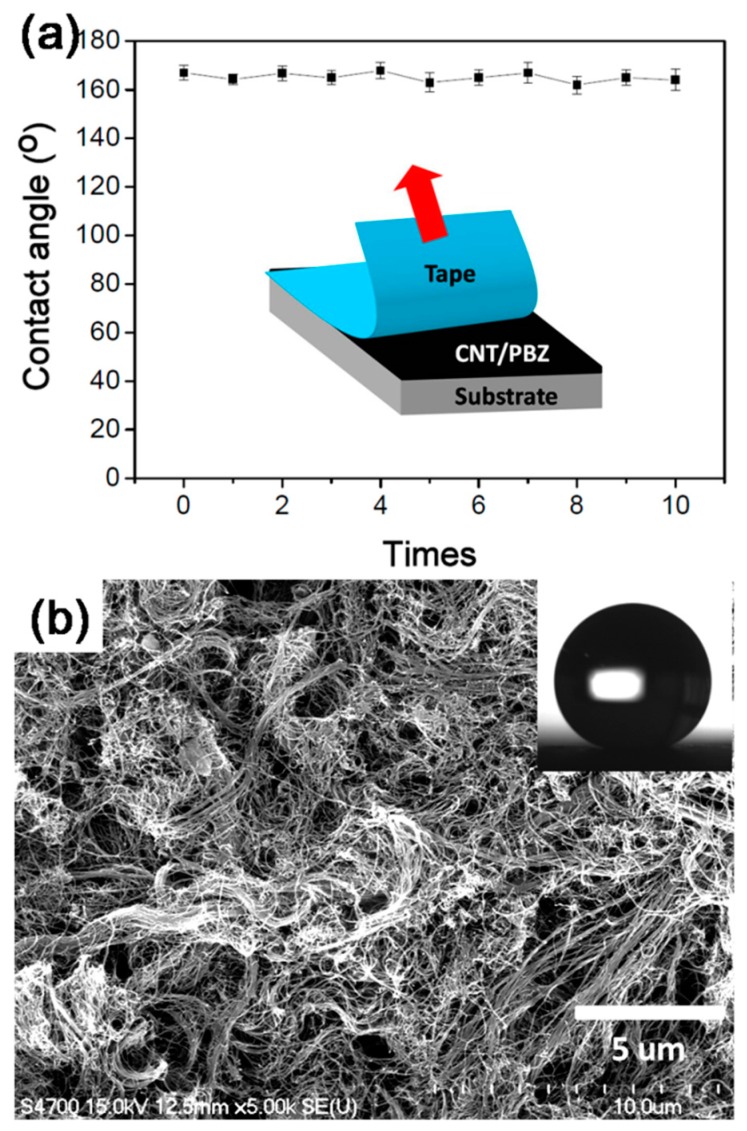
(**a**) Plot of the water contact angles as a function of the number of times of tape test. Inset: schematic of tape test. (**b**) SEM image of the superhydrophobic CNT/polybenzoxazine film after performing the tape test 10 times. Inset: photograph of the water droplet on the sample.

Since sample B showed good film-forming characteristics, higher water contact angle of 167 ± 1°, and smaller sliding angle (lower than 4°), compared to sample A, only the superhydrophobic CNT/polybenzoxazine films synthesized using suspension B were considered for further experiments. To investigate the adhesion of these superhydrophobic CNT/polybenzoxazine films to the substrate, a Scotch tape test was used. For the test, the tape is pressed over the coating and then removed. The water contact angles of the superhydrophobic CNT/polybenzoxazine surface marginally reduced from167° to 164° after 10 cycles of adhesion test (Figure 3a), confirming that the superhydrophobic property of the film remains unaltered. Figure 3b presents the SEM images of the superhydrophobic CNT/polybenzoxazine film after performing the tape test 10 times. Comparing this image with the original film (Figure 2c), it was observed that the surface morphology of the superhydrophobic surface changed only slightly. It indicates that the superhydrophobic CNT/polybenzoxazine film adhered strongly to the substrate and the superhydrophobic property was not destroyed by the tape test.

**Figure 4:**
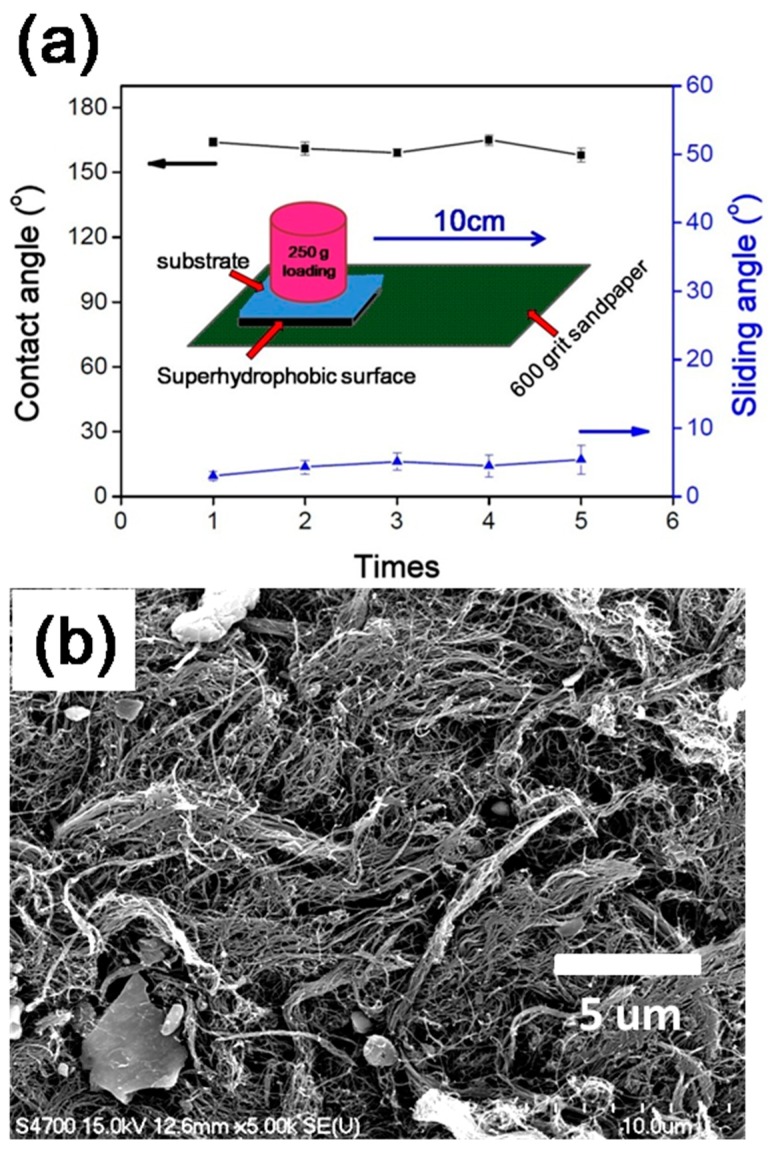
(**a**) Plot of the water contact/sliding angles as a function of the number of times of sandpaper abrasion. Inset: schematic of abrasion test. (**b**) SEM image of the superhydrophobic CNT/polybenzoxazine film after performing sandpaper abrasion after 5 times.

Mechanical damage usually destroy the micro-structures of superhydrophobic surfaces, and limit the pragmatic applications of superhydrophobic films. We studied the mechanical durability of the superhydrophobic CNT/polybenzoxazine films through the linear abrasion test (Figure 4a). The superhydrophobic CNT/polybenzoxazine films, weighing 250 g, were placed facedown to 600 grit sandpaper moved for 10 cm along the ruler; this was termed as one abrasion cycle. Figure 4a shows the water contact angles and sliding angles after each abrasion test. We observed that water contact angles placed on those superhydrophobic CNT/polybenzoxazine films are changing from 165° to 158° after 5 cycles of abrasion, whereas the proportional sliding angles ranged from 3° to 5°, indicating good mechanical abrasion resistance of the superhydrophobic surface. Figure 4b shows the SEM image of the superhydrophobic CNT/polybenzoxazine surface after sandpaper-abrasion tests. Interestingly, the surface of the sample still retained significant amounts of CNT/polybenzoxazine composites even after 5 cycles of sandpaper abrasion; the contact angle was 158°, confirming the robustness and superhydrophobicity of the films.

**Figure 5:**
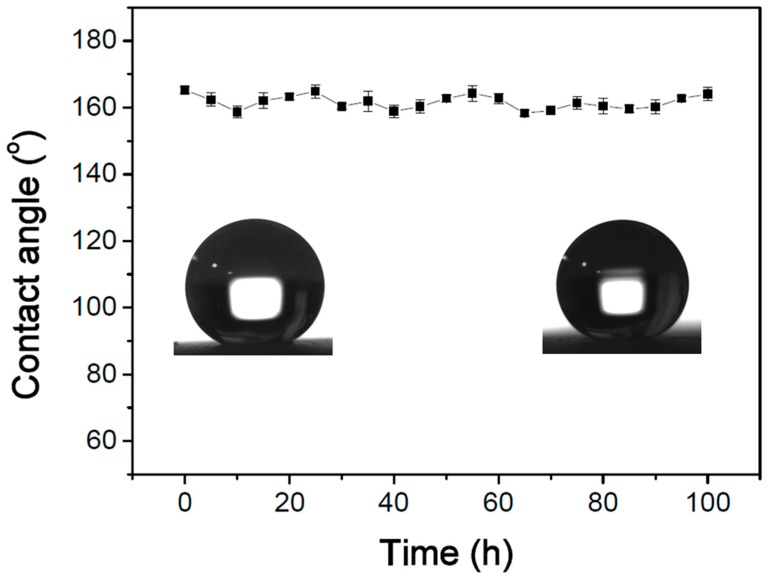
Stability of the superhydrophobic CNT/polybenzoxazine film. The value of contact angle of water as a function of ultraviolet (UV) illumination time.

Besides mechanical stability, UV durability is also an important factor for superhydrophobic films with regard to outdoor applications. Superhydrophobic films are usually prepared by covering the rough substrate with organic coatings that possess low surface-free energy. However, the organic coatings usually deteriorate upon UV exposure, decreasing the self-cleaning properties of superhydrophobic films. Recently, some researchers have developed several UV-durable superhydrophobic materials to address this limitation [22,23,24,25]. Wong and coworkers synthesized durable coatings with superhydrophobicity through template-free micro-nano texturing of interpenetrated polymer networks [22]. Their superhydrophobic coatings possess superior abrasion, UV, and chemical resistance. Ren et al. produced superhydrophobic fabrics through dip coating with ZnO-polydimethylsiloxane (PDMS) followed by thermal treatment [23]. Their ZnO-PDMS coated fabrics possessed UV shielding capacity and maintained superhydrophobicity after UV illumination for up to 75 h. UV-durability of the as-synthesized superhydrophobic CNT/polybenzoxazine film was assessed using a UV lamp at a wavelength of 256 nm. The water contact angle of the superhydrophobic CNT/polybenzoxazine film was measured every 5 h. Upon UV illumination, the values of water contact angle were very stable (Figure 5). Water dropletsexhibiteda spherical shape on superhydrophobic CNT/polybenzoxazine surface, and the water contact angle approximately 164°, even after irradiation by UV light for 100 h; this suggestsexcellent UV-durability of the as-synthesized superhydrophobic films.

**Figure 6:**
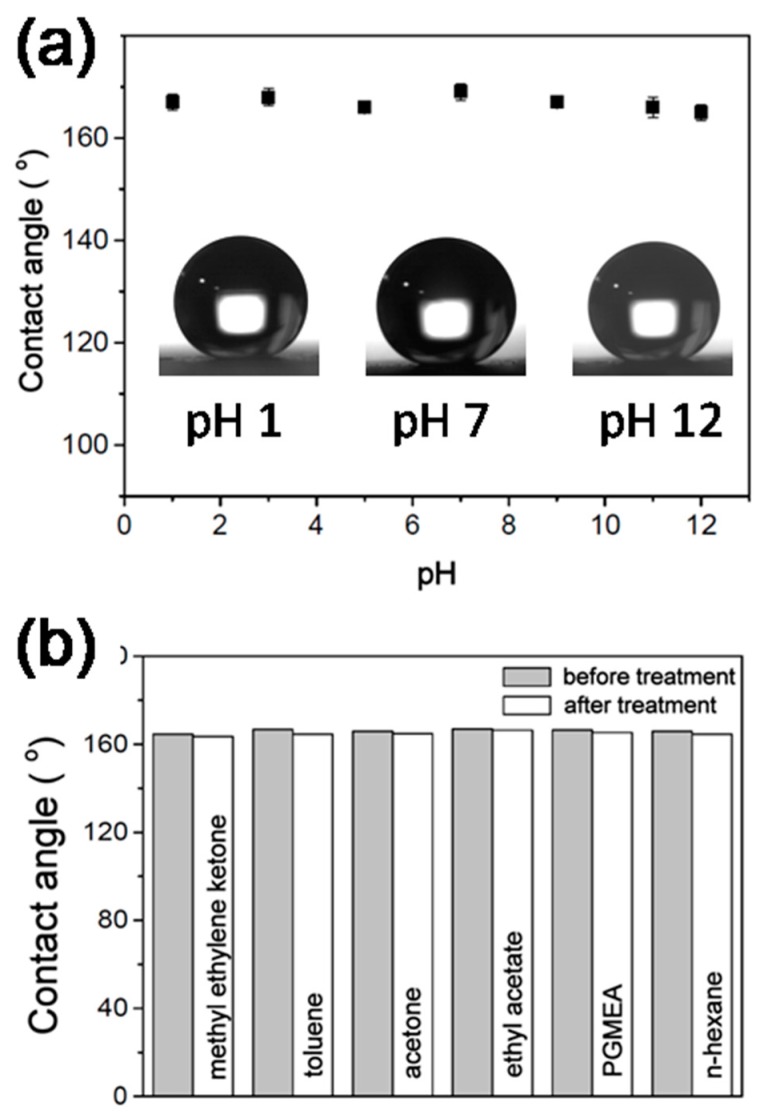
(**a**) Relationship between pH and water contact angle on the superhydrophobic CNT/polybenzoxazine film. (**b**) Durability of the superhydrophobic films after treatment with organic solvents.

These CNT/polybenzoxazine films were very stable under extreme environmental conditions. For practical applications, the thermal stability of the films needs to be considered in superhydrophobic coatings; these should be able to sustain variable and high temperatures. The effect of different thermal treatment durations at 210 °C on the wettability was also systemically studied to estimate the thermal stability of the superhydrophobic CNT/polybenzoxazine films in terms of water contact angles. Figure S2 indicates that increase of treatment time has little or no effect on the film’s water contact angle, substantiating the superhydrophobicity as well as thermal stability of the CNT/polybenzoxazine films. The influence of the different pH of water droplets on the water contact angle is also an important issue to be studied. Figure 6a shows the relationship between the water contact angles on the superhydrophobic CNT/polybenzoxazine surface and the pH value. All the measured contact angles were between approximately 162° and 167° over the entire pH range tested (pH = 1.2–12.3). It revealed that the surfaces exhibited superhydrophobic properties not only for pure water but also under corrosive acidic and basic conditions. For determining the chemical resistance of the as prepared superhydrophobic surfaces, their wettability against organic solvents was evaluated. The contact angles of the superhydrophobic CNT/polybenzoxazine films before and after treatment with various organic solvents were shown in Figure 6b. The contact angle of the films remained nearly unchanged after such treatment.

**Figure 7:**
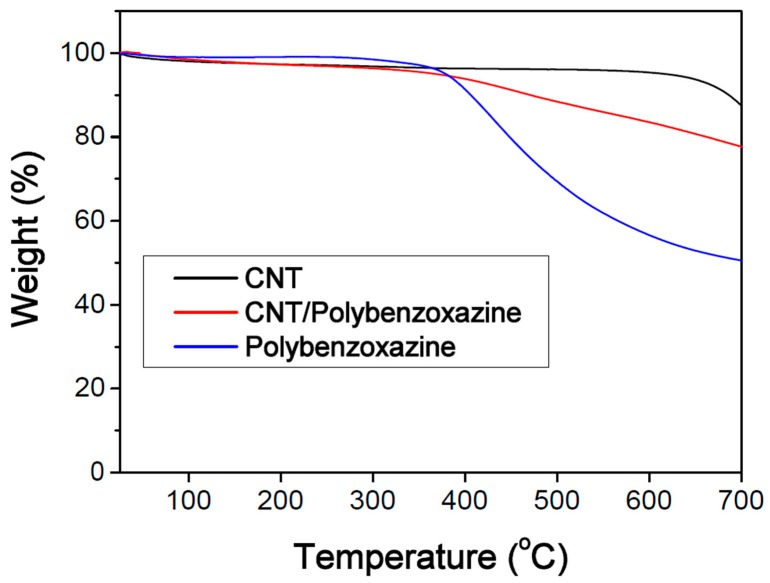
Thermogravimetric analysis (TGA) traces of crude CNTs, polybenzoxazine and the CNT/polybenzoxazine composite under N_2_.

Finally, we used TGA to investigate the thermal properties of the CNT/polybenzoxazine composites. Figure 7 reveals that the decomposition of the CNT/polybenzoxazine composites was delayed relative to that of the pristine polybenzoxazine. The temperatures required for loss of 10 wt % of the CNT/polybenzoxazine composites and polybenzoxazine were 470 and 406 °C, respectively, indicating that the thermal stability of polybenzoxazine was improved after hybridization with CNTs. The char yield of the CNT/polybenzoxazine composite was as high as 78 wt % at 700 °C. Compared with many other kinds of durable superhydrophobic materials, our CNT/polybenzoxazine coatings are easier to prepare and exhibit remarkable durability against thermal treatment, organic solvents, sandpaper abrasion, and UV irradiation, while retaining their superhydrophobicity (Table 1) [10,11,12,13,16].

## 4. Conclusions

We developed a simple and facile method for the fabrication of durable superhydrophobic films from CNT and main-chain type polybenzoxazine precursors. These CNT/polybenzoxazine films were sufficiently robust as thermal treatment, tape test, and sandpaper abrasion processes did not affect their superhydrophobicity. The as-prepared surfaces exhibited superhydrophobic properties not only for pure water but also under corrosive acidic and basic conditions. The non-fluorine superhydrophobic CNT/polybenzoxazine films also possess remarkable stability to organic solvent treatments in terms of the water contact angle. Furthermore, after UV irradiation for 100 h, these CNT/polybenzoxazine films still possessed superhydrophobicity. Our results are significant in terms of their importance to academic research and industrial applications such as dragging reduction, self-cleaning paints preparation, anticorrosive materials fabrication, among others.

## Figures and Tables

**Table 1:** Comparison of various durable superhydrophobic surfaces.

Materials	Water Contact Angle (°)	Water Sliding Angle (°)	Ref.
Perfluorodecyltrimethoxysilane (FAS)-TiO_2_ nanoparticles coated rough electroplated Zn surface	164.1	3.2	[10]
nanoporous silica thin films	162	4	[11]
branched fluorinated epoxy coated PET fabric	169	12	[12]
NiO–SiO_2_–ultra high molecular weight polyethylene coatings	162	3	[13]
UV-curable PDMS and silica particles	160	5	[16]
CNT/polybenzoxazine coatings	167	3	This work

## Supplementary Materials

The following are available online at https://www.mdpi.com/2073-4360/11/7/1183/s1.
