# Supplementary material for: Facile Fabrication of Durable Superhydrophobic Films from Carbon Nanotube/Main-Chain Type Polybenzoxazine Composites
Source: Polymers (Basel). 2019 Jul 14;11(7):1183. doi: 10.3390/polym11071183 (PMC6680462; doi:10.3390/polym11071183)
Supplement: Supplementary file 1 [file polymers-11-01183-s001.pdf]

Supplementary material

Facile Fabrication of Durable Superhydrophobic Films from Carbon Nanotube/Main-Chain Type Polybenzoxazine Composites

Chih-Feng Wang[1]*, Wen-Ning Wang[2], Ching-Hsuan Lin[3]*, Kuo-Jung Lee[2], Chien-Chieh Hu[1], and Juin-Yih Lai[1, 4, 5]

[1]Graduate Institute of Applied Science and Technology, National Taiwan University of Science and Technology, Taipei, 106, Taiwan

[2]Department of Materials Science and Engineering, I-Shou University, Kaohsiung, 840, Taiwan.

[3]Department of Chemical Engineering, National Chung Hsing University, Taichung 402, Taiwan

[4]Department of Chemical Engineering, National Taiwan University of Science and Technology, Taipei 106, Taiwan

[5]R&D Centre for Membrane Technology, Chung Yuan University, Taoyuan 320, Taiwan

*To whom all correspondence should be addressed

cfwang@mail.ntust.edu.tw Tel.: +886-2-27301210; Fax: +886-2-27303733

linch@dragon.nchu.edu.tw Tel: +886-4-22850180; Fax: +886-4-22854734

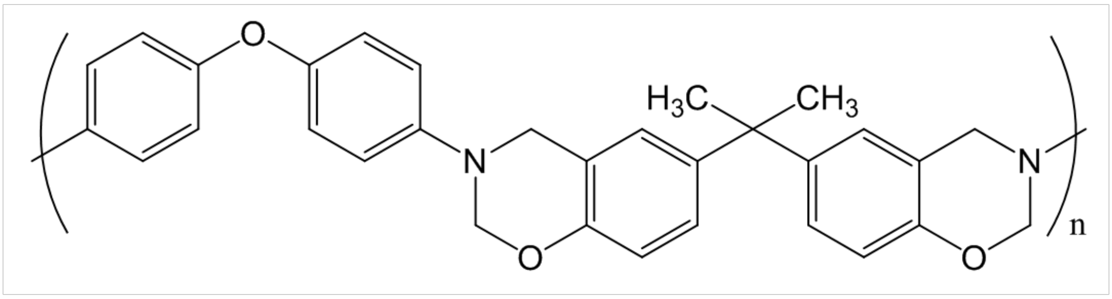

Figure S1. The chemical structure of the main-chain type polybenzoxazine precursor P(B-oda).

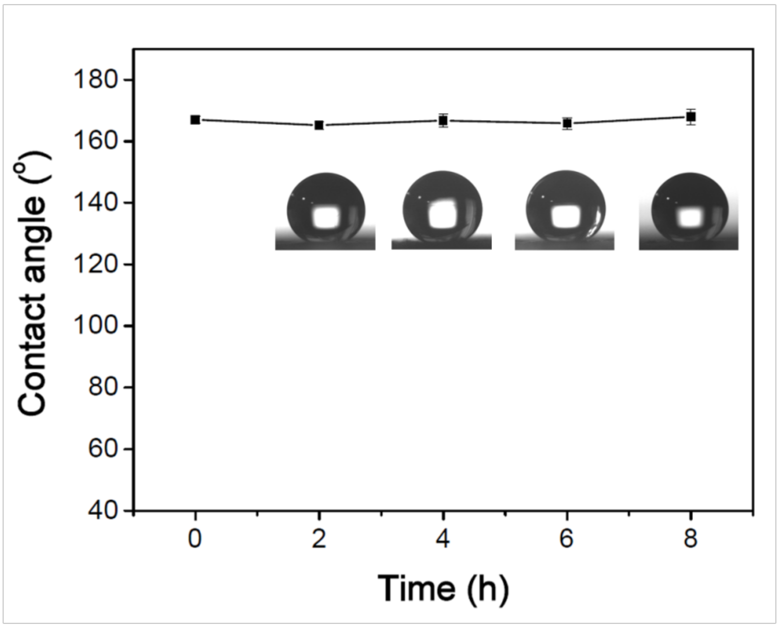

Figure S2. Thermal stability of the superhydrophobic CNT/polybenzoxazine film treated at various time at 210 ^{o}C.
